# Supplementary material for: Association between preterm birth and economic and educational outcomes in adulthood: A population-based matched cohort study
Source: PLoS One. 2024 Nov 6;19(11):e0311895. doi: 10.1371/journal.pone.0311895 (PMC11540172; doi:10.1371/journal.pone.0311895)
Supplement: S1 Table — (DOCX) [file pone.0311895.s001.docx]

**Association between preterm birth and economic and educational outcomes in adulthood: A population-based matched cohort study**

**Authors:** Asma M. Ahmed, Eleanor Pullenayegum, Sarah D. McDonald, Marc Beltempo, Shahirose S. Premji, Jason D. Pole, Fabiana Bacchini, Prakesh S. Shah, Petros Pechlivanoglou,

**S1 Table. Description of different datasets at Statistics Canada used in the study and the relevant study variables.**

| **Name of the dataset** | **Brief description** | **Study variables** |
| --- | --- | --- |
| **The Vital Statistics-Birth (VSB) file** | A cross-sectional administrative database that collects data on all live births in Canada from all provincial and territorial vital statistics registries. | Exposure: gestational age.  Matching variables: individual's sex (female or male), the birth plurality (single or multiple), province of birth, and birth year, mother’s marital status (single, married, other [includes widowed, divorced, separated], or missing), parental age (<20, 20-24, 25-29, 30-34, 35-39, >40 years for the mother and <25, 25–29,30–34, 35–39, >40 years, or missing for the father), parental place of birth based on Statistical Classification of Countries and Areas of Interest for Social Statistics (Canada, North America excluding Canada, Central and South America, Europe, Africa, Asia, and other [Oceania/Antarctica and adjacent islands, missing, or unknown]), and maternal parity (0, 1, 2, 3, or >4 previous live births). |
| **The Vital Statistics- Death (VSD) file** | A cross-sectional, administrative database that collects mortality data from all provincial and territorial vital statistics registries. | Mortality during the time of follow-up. |
| **The TI Family File (TIFF)** | The T1FF includes data on all Canadian residents who filed their tax return (T1 form) or received the Canada Child Tax Benefit in a given year with their spouses and children. The T1FF contains demographic information and data on income and household composition. | Outcomes: Annual employment income; employment.  Matching variables (in sensitivity analysis): family income quintiles at baseline, maternal place of residence (rural/urban). |
| **Postsecondary Student Information System (PSIS)** | A cross-sectional, national database that collects information from Canadian public postsecondary institutions across all provinces and territories. This dataset includes information about the programs and courses offered at these institutions, and data on students enrolled in these institutions (e.g., demographic characteristics and the programs/courses they are enrolled in or graduated from). | Outcome: postsecondary education enrollment and attainment. |
