# Supplementary material for: Association between preterm birth and economic and educational outcomes in adulthood: A population-based matched cohort study
Source: PLoS One. 2024 Nov 6;19(11):e0311895. doi: 10.1371/journal.pone.0311895 (PMC11540172; doi:10.1371/journal.pone.0311895)
Supplement: S2 Table — (DOCX) [file pone.0311895.s002.docx]

**Association between preterm birth and economic and educational outcomes in adulthood: A population-based matched cohort study**

**Authors:** Asma M. Ahmed, Eleanor Pullenayegum, Sarah D. McDonald, Marc Beltempo, Shahirose S. Premji, Jason D. Pole, Fabiana Bacchini, Prakesh S. Shah, Petros Pechlivanoglou,

**S2 Table. Characteristics of individuals included vs excluded from the study population [N (%)].**

| **Characteristics** | **Included sample (n= 2,431,750)** | **Excluded due to missing tax records (n=149,260)** | **SMD** |
| --- | --- | --- | --- |
| Individual’s sex  Female  Male | 1,188,390 (48.9)  1,243,360 (51.1) | 68,140 (45.7)  81,110 (54.3) | 0.06 |
| Maternal parity  >4  0  1  2  3 | 54,290 (2.2)  1,042,670 (42.9)  866,920 (35.6)  358,510 (14.7)  109,370 (4.5) | 5,420 (3.6)  67,220 (45)  47,910 (32.1)  21,000 (14.1)  7,700 (5.2) | 0.11 |
| Maternal age  <20 years  >40 years  20-24 years  25-29 years  30-34 years  35-39 years | 141,640 (5.8)  31,760 (1.3)  466,360 (19.2)  859,170 (35.3)  700,020 (28.8)  232,810 (9.6) | 14,580 (9.8)  2,200 (1.5)  32,650 (21.9)  47,000 (31.5)  38,780 (26)  14,050 (9.4) | 0.18 |
| Paternal age  <25 years  >40 years  25–29 years  30–34 years  35–39 years  Missing | 266,480 (11)  162,530 (6.7)  669,350 (27.5)  787,530 (32.4)  383,080 (15.8)  162,780 (6.7) | 16,320 (10.9)  10,650 (7.1)  33,210 (22.3)  40,640 (27.2)  21,880 (14.7)  26,560 (17.8) | 0.35 |
| Place of birth  Alberta  Atlantic Provinces  British Columbia  Manitoba  Ontario  Quebec  Saskatchewan  Yukon, Nunavut, and Northwest Territory | 262,780 (10.8)  177,660 (7.3)  288,690 (11.9)  106,240 (4.4)  928,180 (38.2)  564,430 (23.2)  93,190 (3.8)  10,580 (0.4) | 13,930 (9.3)  7,870 (5.3)  19,600 (13.1)  5,280 (3.5)  55,720 (37.3)  41,760 (28)  3,520 (2.4)  1,580 (1.1) | 0.18 |
| Marital status  married  missing  Other  Single | 1,669,240 (68.6)  115,970 (4.8)  45,500 (1.9)  601,030 (24.7) | 88,420 (59.2)  11,630 (7.8)  3,300 (2.2)  45,910 (30.8) | 0.21 |
| Birth year  1990  1991  1992  1993  1994  1995  1996 | 351,920 (14.5)  353,230 (14.5)  356,830 (14.7)  346,460 (14.2)  349,240 (14.4)  343,510 (14.1)  330,550 (13.6) | 21,030 (14.1)  22,310 (14.9)  20,830 (14)  20,440 (13.7)  20,100 (13.5)  21,160 (14.2)  23,390 (15.7) | 0.07 |
| Maternal place of birth  Africa  Asia  Canada  Central and South America  Europe  North America  Other | 16,400 (0.7)  123,980 (5.1)  1,962,150 (80.7)  35,670 (1.5)  120,770 (5)  28,760 (1.2)  144,030 (5.9) | 3,240 (2.2)  15,580 (10.4)  84,680 (56.7)  5,920 (4)  12,090 (8.1)  7,990 (5.4)  19,760 (13.2) | 0.55 |
| Paternal place of birth  Africa  Asia  Canada  Central and South America  Europe  North America  Other | 19,190 (0.8)  120,140 (4.9)  1,797,940 (73.9)  35,790 (1.5)  137,860 (5.7)  22,950 (0.9)  297,880 (12.2) | 3,490 (2.3)  15,180 (10.2)  65,000 (43.6)  5,180 (3.5)  12,030 (8.1)  6,750 (4.5)  41,620 (27.9) | 0.67 |
| Birth plurality  Singleton  Multiple | 2,381,740 (97.9)  50,000 (2.1) | 141,530 (94.8)  7,730 (5.2) | 0.17 |
| Preterm birth  Preterm 24-36  Term 37-41 | 172,690 (7.1)  2,259,060 (92.9) | 15,210 (10.2)  134,040 (89.8) | 0.11 |
| Gestational age categories  37-41  34-36  32-33  28-31  24-27 | 2,259,060 (92.9)  131,960 (5.4)  19,870 (0.8)  14,460 (0.6)  6,410 (0.3) | 134,040 (89.8)  10,750 (7.2)  1,870 (1.2)  1,620 (1.1)  980 (0.7) | 0.12 |

Note: All numbers were rounded to the nearest ten for confidentiality reasons
