# Supplementary material for: Association between preterm birth and economic and educational outcomes in adulthood: A population-based matched cohort study
Source: PLoS One. 2024 Nov 6;19(11):e0311895. doi: 10.1371/journal.pone.0311895 (PMC11540172; doi:10.1371/journal.pone.0311895)
Supplement: S3 Table — Characteristics of the unmatched (a) and matched cohorts (b) according to gestational age category [N (%). (DOCX) [file pone.0311895.s003.docx]

**Association between preterm birth and economic and educational outcomes in adulthood: A population-based matched cohort study**

**Authors:** Asma M. Ahmed, Eleanor Pullenayegum, Sarah D. McDonald, Marc Beltempo, Shahirose S. Premji, Jason D. Pole, Fabiana Bacchini, Prakesh S. Shah, Petros Pechlivanoglou,

**S3 Table. Characteristics of the unmatched (a) and matched cohorts (b) according to gestational age category [N (%)].**

**(a) Before matching**

|  | **37-41 weeks (reference, n=2,259,060)** | **34-36 weeks w (n=131,960)** | | **32-33 weeks (n=19,870)** | | **28-31 weeks (n=14,460)** | | **24-27 weeks (n=6,410)** | |
| --- | --- | --- | --- | --- | --- | --- | --- | --- | --- |
| **Characteristics** | **N (%)** | **N (%)** | **SMD** | **N (%)** | **SDM** | **N (%)** | **SDM** | **N (%)** | **SDM** |
| Individual’s sex  Female  Male | 1,109,170 (49.1)  1,149,890 (51.9) | 60,690 (46.0)  71,270 (54.0) | 0.06 | 9,030 (45.4)  10,840 (54.6) | 0.07 | 6,560 (45.4)  7,900 (54.6) | 0.08 | 2,960 (46.1)  3,450 (53.9) | 0.06 |
| Birth plurality  Singleton  Multiple | 2,232,930 (98.8)  26,130 (1.2) | 116,440 (88.2)  15,520 (11.8) | 0.44 | 15,890 (80)  3,980 (20) | 0.64 | 11,350 (78.5)  3,100 (21.5) | 0.68 | 5,140 (80.1)  1,270 (19.9) | 0.64 |
| Maternal parity  >4  0  1  2  3 | 49,230 (2.2)  963,480 (42.6)  812,090 (35.9)  333,710 (14.8)  100,560 (4.5) | 3,800 (2.9)  60,160 (45.6)  42,120 (31.9)  19,120 (14.5)  6,770 (5.1) | 0.10 | 620 (3.1)  9,240 (46.5)  6,190 (31.1)  2,870 (14.4)  960 (4.8) | 0.12 | 470 (3.2)  6,770 (46.8)  4,510 (31.2)  1,960 (13.5)  750 (5.2) | 0.13 | 180 (2.7)  3,030 (47.3)  2,010 (31.4)  850 (13.2)  340 (5.3) | 0.12 |
| Maternal age  <20 years  >40 years  20-24 years  25-29 years  30-34 years  35-39 years | 129,490 (5.7)  28,600 (1.3)  432,630 (19.2)  802,860 (35.5)  651,980 (28.9)  213,500 (9.5) | 8,890 (6.7)  2,370 (1.8)  25,880 (19.6)  43,460 (32.9)  36,740 (27.8)  14,620 (11.1) | 0.09 | 1,480 (7.4)  390 (2)  3,850 (19.4)  6,330 (31.9)  5,560 (28)  2,250 (11.3) | 0.12 | 1,170 (8.1)  290 (2)  2,780 (19.2)  4,550 (31.4)  4,050 (28)  1,640 (11.3) | 0.14 | 610 (9.6)  110 (1.7)  1,230 (19.2)  1,970 (30.8)  1,690 (26.4)  790 (12.4) | 0.19 |
| Paternal age  <25 years  >40 years  25–29 years  30–34 years  35–39 years  Missing | 245,940 (10.9)  149,200 (6.6)  624,360 (27.6)  735,760 (32.6)  355,840 (15.8)  147,960 (6.5) | 15,310 (11.6)  10,150 (7.7)  34,610 (26.2)  39,930 (30.3)  21,000 (15.9)  10,960 (8.3) | 0.09 | 2,460 (12.4)  1,550 (7.8)  5,170 (26)  5,840 (29.4)  3,040 (15.3)  1,810 (9.1) | 0.13 | 1,880 (13)  1,130 (7.8)  3,660 (25.3)  4,180 (28.9)  2,230 (15.4)  1,370 (9.5) | 0.15 | 890 (13.9)  490 (7.7)  1,550 (24.2)  1,830 (28.5)  980 (15.2)  680 (10.5) | 0.20 |
| Maternal place of birth  Africa  Asia  Canada  Central and South America  Europe  North America  Other | 15,010 (0.7)  112,720 (5)  1,830,750 (81)  32,300 (1.4)  112,330 (5)  26,970 (1.2)  128,990 (5.7) | 1,080 (0.8)  9,130 (6.9)  99,330 (75.3)  2,510 (1.9)  6,510 (4.9)  1,350 (1)  12,060 (9.1) | 0.17 | 130 (0.7)  1,160 (5.8)  15,670 (78.9)  400 (2)  910 (4.6)  200 (1)  1,400 (7) | 0.09 | 110 (0.8)  700 (4.8)  11,440 (79.1)  300 (2.1)  700 (4.8)  170 (1.2)  1,040 (7.2) | 0.08 | 70 (1.1)  280 (4.3)  4,970 (77.5)  160 (2.5)  320 (5)  70 (1.1)  540 (8.5) | 0.15 |
| Paternal place of birth  Africa  Asia  Canada  Central and South America  Europe  North America  Other | 17,650 (0.8)  109,490 (4.8)  1,679,970 (74.4)  32,580 (1.4)  128,490 (5.7)  21,470 (1)  269,400 (11.9) | 1,190 (0.9)  8,590 (6.5)  89,490 (67.8)  2,390 (1.8)  7,250 (5.5)  1,120 (0.8)  21,930 (16.6) | 0.17 | 140 (0.7)  1,110 (5.6)  13,990 (70.4)  380 (1.9)  1,030 (5.2)  190 (0.9)  3,030 (15.3) | 0.12 | 140 (0.9)  660 (4.6)  10,180 (70.4)  290 (2)  760 (5.3)  110 (0.8)  2,320 (16.1) | 0.13 | 70 (1)  290 (4.5)  4,320 (67.5)  150 (2.4)  320 (5.1)  60 (1.0)  1,190 (18.6) | 0.21 |
| Maternal marital status at birth  Married  Other  Missing  Single | 1,560,010 (69.1)  103,550 (4.6)  41,770 (1.8)  553,730 (24.5) | 84,430 (64)  8,910 (6.8)  2,840 (2.1)  35,770 (27.1) | 0.12 | 12,230 (61.6)  1,590 (8)  430 (2.2)  5,620 (28.3) | 0.18 | 8,800 (60.8)  1,270 (8.8)  320 (2.2)  4,080 (28.2) | 0.21 | 3,770 (58.8)  650 (10.1)  150 (2.3)  1,840 (28.7) | 0.26 |
| Place of birth  Alberta  Atlantic Provinces  British Columbia  Manitoba  Ontario  Quebec  Saskatchewan  Territories | 244,480 (10.8)  165,250 (7.3)  270,590 (12)  98,290 (4.4)  854,880 (37.8)  528,470 (23.4)  87,150 (3.9)  9,940 (0.4) | 13,530 (10.2)  9,290 (7)  13,400 (10.2)  5,910 (4.5)  56,270 (42.6)  28,510 (21.6)  4,510 (3.4)  550 (0.4) | 0.11 | 2,260 (11.4)  1,480 (7.4)  2,280 (11.5)  950 (4.8)  8,330 (41.9)  3,820 (19.2)  710 (3.5)  50 (0.2) | 0.12 | 1,720 (11.9)  1,160 (8)  1,670 (11.6)  750 (5.2)  5,940 (41)  2,640 (18.2)  560 (3.9)  30 (0.2) | 0.14 | 790 (12.4)  500^a^ (7.7)  760 (11.8)  340 (5.3)  2,760 (43.1)  1,000 (15.6)  260 (4.1)  500^a^ (7.7) | 0.22 |
| Birth year  1990  1991  1992  1993  1994  1995  1996 | 329,490 (14.6)  329,850 (14.6)  332,890 (14.7)  321,780 (14.2)  323,650 (14.3)  316,990 (14)  304,400 (13.5) | 16,780 (12.7)  17,740 (13.4)  18,150 (13.8)  18,920 (14.3)  19,580 (14.8)  20,560 (15.6)  20,230 (15.3) | 0.09 | 2,800 (14.1)  2,670 (13.4)  2,780 (14)  2,840 (14.3)  2,950 (14.8)  2,940 (14.8)  2,880 (14.5) | 0.05 | 1,990 (13.8)  2,100 (14.5)  2,070 (14.3)  2,010 (13.9)  2,120 (14.7)  2,070 (14.3)  2,090 (14.5) | 0.04 | 860 (13.5)  880 (13.7)  930 (14.5)  910 (14.2)  940 (14.6)  950 (14.8)  950 (14.8) | 0.06 |

Note: All numbers were rounded to the nearest ten for confidentiality reasons

^a^ Atlantic provinces and Territories were combined due to small cell count

**(b) After matching [weighted]**

|  | **34-36 weeks** | | **32-33 weeks** | | **28-31 weeks** | | **24-27 weeks** | |
| --- | --- | --- | --- | --- | --- | --- | --- | --- |
| **Characteristics** | **Term (n=1,854,780)** | **Preterm (n=121,430)** | **Term (n=1,310,980)** | **Preterm (n=17,690)** | **Term (n=1,187,270)** | **Preterm (n=12,780)** | **Term (n=843,670)** | **Preterm (n=5,600)** |
| Individual’s sex  Female  Male | 849,410 (45.8)  1,005,370 (54.2) | 55,610 (45.8)  65,820 (54.2) | 591,720 (45.1)  719,260 (54.9) | 7,980 (45.1)  9,710 (54.9) | 537,430 (45.3)  649,840 (54.7) | 5,790 (45.3)  7,000 (54.7) | 390,790 (46.3)  452,880 (53.7) | 2,590 (46.3)  3,010 (53.7) |
| Birth plurality  Singleton  Multiple | 1,705,690 (92)  149,090 (8) | 111,670 (92)  9,760 (8) | 1,125,850 (85.9)  185,130 (14.1) | 15,190 (85.9)  2,500 (14.1) | 1,007,700 (84.9)  179,580 (15.1) | 10,850 (84.9)  1,930 (15.1) | 728,830 (86.4)  114,840 (13.6) | 4,840 (86.4)  760 (13.6) |
| Maternal parity  >4  0  1  2  3 | 41,640 (2.2)  874,750 (47.2)  598,160 (32.2)  258,350 (13.9)  81,880 (4.4) | 2,730 (2.2)  57,270 (47.2)  39,160 (32.2)  16,910 (13.9)  5,360 (4.4) | 30,240 (2.3)  636,920 (48.6)  410,210 (31.3)  180,690 (13.8)  52,920 (4) | 410 (2.3)  8,590 (48.6)  5,540 (31.3)  2,440 (13.8)  710 (4) | 26,850 (2.3)  583,320 (49.1)  371,880 (31.3)  152,640 (12.9)  52,580 (4.4) | 290 (2.3)  6,280 (49.1)  4,000 (31.3)  1,640 (12.9)  570 (4.4) | 16,880 (2)  414,450 (49.1)  266,450 (31.6)  107,910 (12.8)  37,980 (4.5) | 110 (2)  2,750 (49.1)  1,770 (31.6)  720 (12.8)  250 (4.5) |
| Maternal age  <20 years  >40 years  20-24 years  25-29 years  30-34 years  35-39 years | 126,210 (6.8)  24,710 (1.3)  367,940 (19.8)  624,710 (33.7)  518,460 (28)  192,740 (10.4) | 8,260 (6.8)  1,620 (1.3)  24,090 (19.8)  40,900 (33.7)  33,940 (28)  12,620 (10.4) | 98,570 (7.5)  18,230 (1.4)  258,360 (19.7)  431,560 (32.9)  367,970 (28.1)  136,290 (10.4) | 1,330 (7.5)  250 (1.4)  3,490 (19.7)  5,820 (32.9)  4,970 (28.1)  1,840 (10.4) | 98,100 (8.3)  16,070 (1.4)  229,840 (19.4)  384,700 (32.4)  333,240 (28.1)  125,320 (10.6) | 1,060 (8.3)  170 (1.4)  2,470 (19.4)  4,140 (32.4)  3,590 (28.1)  1,350 (10.6) | 81,840 (9.7)  9,340 (1.1)  160,660 (19)  269,320 (31.9)  225,460 (26.7)  97,060 (11.5) | 540 (9.7)  60 (1.1)  1,070 (19)  1,790 (31.9)  1,500 (26.7)  640 (11.5) |
| Paternal age  <25 years  >40 years  25–29 years  30–34 years  35–39 years  Missing | 214,740 (11.6)  127,740 (6.9)  497,310 (26.8)  574,410 (31)  289,510 (15.6)  151,080 (8.1) | 14,060 (11.6)  8,360 (6.9)  32,560 (26.8)  37,610 (31)  18,950 (15.6)  9,890 (8.1) | 163,420 (12.5)  90,420 (6.9)  350,630 (26.7)  392,200 (29.9)  199,220 (15.2)  115,100 (8.8) | 2,210 (12.5)  1,220 (6.9)  4,730 (26.7)  5,290 (29.9)  2,690 (15.2)  1,550 (8.8) | 157,190 (13.2)  79,520 (6.7)  310,100 (26.1)  355,440 (29.9)  179,210 (15.1)  105,810 (8.9) | 1,690 (13.2)  860 (6.7)  3,340 (26.1)  3,830 (29.9)  1,930 (15.1)  1,140 (8.9) | 115,140 (13.6)  61,040 (7.2)  209,030 (24.8)  248,370 (29.4)  125,240 (14.8)  84,850 (10.1) | 760 (13.6)  410 (7.2)  1,390 (24.8)  1,650 (29.4)  830 (14.8)  560 (10.1) |
| Maternal place of birth  Africa  Asia  Canada  Central and South America  Europe  North America  Other | 10,340 (0.6)  121,920 (6.6)  1,436,010 (77.4)  27,630 (1.5)  80,480 (4.3)  12,010 (0.6)  166,400 (9) | 680 (0.6)  7,980 (6.6)  94,020 (77.4)  1,810 (1.5)  5,270 (4.3)  790 (0.6)  10,890 (9) | 5,190 (0.4)  70,110 (5.3)  1,069,150 (81.6)  20,600 (1.6)  48,620 (3.7)  9,120 (0.7)  88,190 (6.7) | 70 (0.4)  950 (5.3)  14,430 (81.6)  280 (1.6)  660 (3.7)  120 (0.7)  1,190 (6.7) | 6,970 (0.6)  48,960 (4.1)  976,110 (82.2)  19,140 (1.6)  47,290 (4)  7,900 (0.7)  80,920 (6.8) | 80 (0.6)  530 (4.1)  10,510 (82.2)  210 (1.6)  510 (4)  90 (0.7)  870 (6.8) | 5,270 (0.6)  34,510 (4.1)  679,250 (80.5)  16,880 (2)  34,210 (4.1)  5,880 (0.7)  67,670 (8) | 40 (0.6)  230 (4.1)  4,510 (80.5)  110 (2)  230 (4.1)  40 (0.7)  450 (8) |
| Paternal place of birth  Africa  Asia  Canada  Central and South America  Europe  North America  Other | 11,260 (0.6)  115,950 (6.3)  1,296,530 (69.9)  26,650 (1.4)  90,770 (4.9)  9,840 (0.5)  303,790 (16.4) | 740 (0.6)  7,590 (6.3)  84,880 (69.9)  1,750 (1.4)  5,940 (4.9)  640 (0.5)  19,890 (16.4) | 5,110 (0.4)  67,740 (5.2)  961,610 (73.4)  19,860 (1.5)  56,180 (4.3)  6,820 (0.5)  193,660 (14.8) | 70 (0.4)  910 (5.2)  12,980 (73.4)  270 (1.5)  760 (4.3)  90 (0.5)  2,610 (14.8) | 7,800 (0.7)  47,190 (4)  872,430 (73.5)  18,390 (1.5)  51,930 (4.4)  6,130 (0.5)  183,390 (15.4) | 80 (0.7)  510 (4)  9,390 (73.5)  200 (1.5)  560 (4.4)  70 (0.5)  1,970 (15.4) | 4,970 (0.6)  32,700 (3.9)  598,170 (70.9)  16,130 (1.9)  37,980 (4.5)  3,920 (0.5)  149,810 (17.8) | 30 (0.6)  220 (3.9)  3,970 (70.9)  110 (1.9)  250 (4.5)  1020 (18.3)^a^  1020 (18.3)^a^ |
| Marital status  Married  Other  Missing  Single | 1,205,510 (65)  117,110 (6.3)  29,170 (1.6)  502,990 (27.1) | 78,930 (65)  7,670 (6.3)  1,910 (1.6)  32,930 (27.1) | 823,690 (62.8)  96,120 (7.3)  20,160 (1.5)  371,010 (28.3) | 11,110 (62.8)  1,300 (7.3)  270 (1.5)  5,010 (28.3) | 741,160 (62.4)  95,220 (8)  18,210 (1.5)  332,680 (28) | 7,980 (62.4)  1,030 (8)  200 (1.5)  3,580 (28) | 510,000 (60.5)  77,310 (9.2)  13,560 (1.6)  242,790 (28.8) | 3,380 (60.5)  510 (9.2)  90 (1.6)  1,610 (28.8) |
| Place of birth  Alberta  Atlantic Provinces  British Columbia  Manitoba  Ontario  Quebec  Saskatchewan Territories | 184,020 (9.9)  129,890 (7)  179,730 (9.7)  77,300 (4.2)  807,010 (43.5)  409,740 (22.1)  60,730 (3.3)  6,350 (0.3) | 12,050 (9.9)  8,500 (7)  11,770 (9.7)  5,060 (4.2)  52,840 (43.5)  26,830 (22.1)  3,980 (3.3)  420 (0.3) | 145,480 (11.1)  96,050 (7.3)  141,560 (10.8)  58,400 (4.5)  563,330 (43)  258,650 (19.7)  44,760 (3.4)  2,740 (0.2) | 1,960 (11.1)  1340 (7.5)^b^  1,910 (10.8)  790 (4.5)  7,600 (43)  3,490 (19.7)  600 (3.4)  1340 (7.5)^b^ | 137,770 (11.6)  95,320 (8)  130,900 (11)  56,860 (4.8)  495,630 (41.7)  222,780 (18.8)  45,890 (3.9)  2,140 (0.2) | 1,480 (11.6)  1050 (8.2)^b^  1,410 (11)  610 (4.8)  5,340 (41.7)  2,400 (18.8)  490 (3.9)  1050 (8.2)^b^ | 102,930 (12.2)  65,110 (7.7)  95,700 (11.3)  43,560 (5.2)  368,640 (43.7)  133,230 (15.8)  33,610 (4)  900 (0.1) | 680 (12.2)  440 (7.8)^b^  640 (11.3)  290 (5.2)  2,450 (43.7)  880 (15.8)  220 (4)  440 (7.8)^b^ |
| Birth year  1990  1991  1992  1993  1994  1995  1996 | 238,510 (12.9)  250,240 (13.5)  256,620 (13.8)  266,410 (14.4)  273,770 (14.8)  287,520 (15.5)  281,720 (15.2) | 15,620 (12.9)  16,380 (13.5)  16,800 (13.8)  17,440 (14.4)  17,920 (14.8)  18,820 (15.5)  18,440 (15.2) | 185,950 (14.2)  177,800 (13.6)  184,760 (14.1)  188,910 (14.4)  194,470 (14.8)  192,540 (14.7)  186,540 (14.2) | 2,510 (14.2)  2,400 (13.6)  2,490 (14.1)  2,550 (14.4)  2,620 (14.8)  2,600 (14.7)  2,520 (14.2) | 169,730 (14.3)  173,260 (14.6)  175,300 (14.8)  164,340 (13.8)  171,030 (14.4)  168,710 (14.2)  164,900 (13.9) | 1,830 (14.3)  1,870 (14.6)  1,890 (14.8)  1,770 (13.8)  1,840 (14.4)  1,820 (14.2)  1,780 (13.9) | 118,610 (14.1)  115,740 (13.7)  123,880 (14.7)  120,120 (14.2)  124,030 (14.7)  121,770 (14.4)  119,510 (14.2) | 790 (14.1)  770 (13.7)  820 (14.7)  800 (14.2)  820 (14.7)  810 (14.4)  790 (14.2) |

Note: All numbers were rounded to the nearest ten for confidentiality reasons

^a^ North America and other categories were combined due to small cell count

^b^ Atlantic provinces and territories were combined due to small cell count
