# Supplementary material for: Association between preterm birth and economic and educational outcomes in adulthood: A population-based matched cohort study
Source: PLoS One. 2024 Nov 6;19(11):e0311895. doi: 10.1371/journal.pone.0311895 (PMC11540172; doi:10.1371/journal.pone.0311895)
Supplement: S4 Table — (DOCX) [file pone.0311895.s004.docx]

**Association between preterm birth and economic and educational outcomes in adulthood: A population-based matched cohort study**

**Authors:** Asma M. Ahmed, Eleanor Pullenayegum, Sarah D. McDonald, Marc Beltempo, Shahirose S. Premji, Jason D. Pole, Fabiana Bacchini, Prakesh S. Shah, Petros Pechlivanoglou,

**S4 Table. Descriptive statistics for study outcomes by preterm birth (and gestational age categories) in the unmatched cohorts.**

**(a) Economic outcomes**

|  | **Age 18-22 years** | | **Age 23-28 years** | |
| --- | --- | --- | --- | --- |
| Gestational age at birth | **Mean income per year (SD)** | **% employed** | **Mean income per year (SD)** | **% employed** |
| Term (37-41 weeks) | 12,900 (15,200) | 81% | 30,600 (32,300) | 89% |
| Preterm 24-36 weeks | 11,900 (14,000) | 76% | 28,400 (25,800) | 86% |
| Preterm 34-36 weeks | 12,100 (14,100) | 77% | 28,900 (26,200) | 87% |
| Preterm 32-33 weeks | 11,900 (14,000) | 76% | 28,400 (25,900) | 86% |
| Preterm 28-31 weeks | 11,000 (13,700) | 72% | 25,800 (24,600) | 83% |
| Preterm 24-27 weeks | 8,300 (11,900) | 62% | 21,100 (22,400) | 75% |

Note: All numbers were rounded to the nearest hundred for confidentiality reasons

**(b) Educational outcomes**

|  | **Enrollment at age 18-22 years** | | **Graduation at age 22-27 years** | | |
| --- | --- | --- | --- | --- | --- |
| Gestational age at birth | **% enrolled in college** | **% enrolled in university** | **% graduated with non- university degree** | **% graduated with a university degree** | **% graduated with a postgraduate degree** |
| Term (37-41 weeks) | 27% | 40% | 20% | 21% | 2% |
| Preterm 24-36 weeks | 28% | 37% | 19% | 19% | 2% |
| Preterm 34-36 weeks | 28% | 38% | 19% | 19% | 2% |
| Preterm 32-33 weeks | 28% | 36% | 19% | 19% | 2% |
| Preterm 28-31 weeks | 28% | 33% | 19% | 17% | 2% |
| Preterm 24-27 weeks | 30% | 28% | 19% | 14% | 1% |
