# Supplementary material for: Association between preterm birth and economic and educational outcomes in adulthood: A population-based matched cohort study
Source: PLoS One. 2024 Nov 6;19(11):e0311895. doi: 10.1371/journal.pone.0311895 (PMC11540172; doi:10.1371/journal.pone.0311895)
Supplement: S5 Table — Associations between preterm birth and employment income and employment per year, at or after the age of 18 years for individuals born in 1990–1996 in Canada, stratified by age group (a) and birth cohort (b). (DOCX) [file pone.0311895.s005.docx]

**Association between preterm birth and economic and educational outcomes in adulthood: A population-based matched cohort study**

**Authors:** Asma M. Ahmed, Eleanor Pullenayegum, Sarah D. McDonald, Marc Beltempo, Shahirose S. Premji, Jason D. Pole, Fabiana Bacchini, Prakesh S. Shah, Petros Pechlivanoglou,

**S5 Table. Associations between preterm birth and employment income and employment per year, at or after the age of 18 years for individuals born in 1990-1996 in Canada, stratified by age group (a) and birth cohort (b).**

**(a) Stratified by age group**

|  | **Age 18-22 years** | | | **Age 23-25 years** | | | **Age 26-28 years** | | |
| --- | --- | --- | --- | --- | --- | --- | --- | --- | --- |
|  | **Mean income differences (95% CI)** | | | | | | | | |
| Gestational age | **Unmatched** | **Matched model 1** | **Matched model 2** | **Unmatched** | **Matched model 1** | **Matched model 2** | **Unmatched** | **Matched model 1** | **Matched model 2** |
| 24-36 w  34-36 w  32-33 w  28-31 w  24-27 w  37-41 w | -904  (-1014, -904)  -679  (-803, -679)  -823  (-1135, -823)  -1691  (-2057, -1691)  -4247  (-4807, -4247)  Ref. | -609  (-676, -542)  -375  (-450, -300)  -725  (-902, -548)  -1671  (-1880, -1461)  -4030  (-4352, -3707)  Ref. | -621  (-695, -548)  -380  (-462, -297)  -734  (-931, -536)  -1694  (-1930, -1459)  -4115  (-4485, -3745)  Ref. | -1738  (-1975, -1738)  -1284  (-1549, -1284)  -1496  (-2179, -1496)  -3609  (-4379, -3609)  -7704  (-8952, -7704)  Ref. | -1445  (-1628, -1262)  -1028  (-1247, -808)  -1480  (-1860, -1101)  -3635  (-4069, -3201)  -7481  (-8182, -6780)  Ref. | -1455  (-1641, -1269)  -1032  (-1255, -809)  -1475  (-1863, -1086)  -3653  (-4095, -3210)  -7547  (-8264, -6829)  Ref. | -2172  (-2627, -2172)  -1429  (-1948, -1429)  -1981  (-3312, -1981)  -5024  (-6424, -5024)  -10059  (-12332, -10059)  Ref. | -2198  (-2580, -1817)  -1583  (-2054, -1112)  -2169  (-2924, -1414)  -5497  (-6285, -4709)  -10262  (-11525, -8998)  Ref. | -2202  (-2583, -1820)  -1584  (-2055, -1112)  -2167  (-2924, -1411)  -5509  (-6299, -4718)  -10325  (-11591, -9059)  Ref. |
|  | **Ratios of income (95% CI)** | | | | | | | | |
| Gestational age | **Unmatched** | **Matched model 1** | **Matched model 2** | **Unmatched** | **Matched model 1** | **Matched model 2** | **Unmatched** | **Matched model 1** | **Matched model 2** |
| 24-36 w  34-36 w  32-33 w  28-31 w  24-27 w  37-41 w | 0.93 (0.92, 0.93)  0.94 (0.94, 0.95)  0.92 (0.91, 0.94)  0.86 (0.84, 0.87)  0.65 (0.63, 0.67)  Ref. | 0.95  (0.95, 0.96)  0.97  (0.96, 0.98)  0.94  (0.93, 0.96)  0.87  (0.85, 0.89)  0.68  (0.66, 0.71)  Ref. | 0.96  (0.95, 0.96)  0.97  (0.97, 0.98)  0.95  (0.93, 0.96)  0.88  (0.86, 0.9)  0.71  (0.68, 0.74)  Ref. | 0.93  (0.93, 0.94)  0.95  (0.94, 0.95)  0.93  (0.92, 0.94)  0.85  (0.84, 0.87)  0.69  (0.67, 0.72)  Ref. | 0.95 (0.94, 0.95)  0.96 (0.95, 0.97)  0.94 (0.93, 0.96)  0.87 (0.85, 0.88)  0.72 (0.69, 0.74)  Ref. | 0.94  (0.93, 0.95)  0.96  (0.94, 0.97)  0.94  (0.93, 0.96)  0.87  (0.85, 0.88)  0.72  (0.7, 0.75)  Ref. | 0.94  (0.93, 0.94)  0.95  (0.95, 0.96)  0.93  (0.91, 0.95)  0.85  (0.83, 0.86)  0.7  (0.67, 0.73)  Ref. | 0.94  (0.93, 0.95)  0.96  (0.95, 0.97)  0.94  (0.92, 0.96)  0.85  (0.83, 0.87)  0.72  (0.69, 0.75)  Ref. | 0.94  (0.93, 0.96)  0.96  (0.94, 0.98)  0.94  (0.92, 0.96)  0.86  (0.83, 0.88)  0.71  (0.68, 0.75)  Ref. |
|  | **Risk ratios for employment (95% CI)** | | | | | | | | |
| Gestational age | **Unmatched** | **Matched model 1** | **Matched model 2** | **Unmatched** | **Matched model 1** | **Matched model 2** | **Unmatched** | **Matched model 1** | **Matched model 2** |
| 24-36 w  34-36 w  32-33 w  28-31 w  24-27 w  37-41 w | 0.95  (0.94, 0.95)  0.96  (0.96, 0.96)  0.94  (0.94, 0.95)  0.89  (0.88, 0.9)  0.77  (0.76, 0.79)  Ref. | 0.96  (0.96, 0.97)  0.98  (0.97, 0.98)  0.96  (0.95, 0.96)  0.91  (0.9, 0.92)  0.8  (0.79, 0.82)  Ref. | 0.98  (0.98, 0.98)  0.99  (0.98, 0.99)  0.97  (0.96, 0.98)  0.93  (0.92, 0.94)  0.83  (0.82, 0.85)  Ref. | 0.97  (0.97, 0.97)  0.98  (0.98, 0.98)  0.96  (0.96, 0.97)  0.93  (0.92, 0.93)  0.84  (0.83, 0.85)  Ref. | 0.98  (0.97, 0.98)  0.98  (0.98, 0.99)  0.97  (0.96, 0.98)  0.94  (0.93, 0.94)  0.86  (0.84, 0.87)  Ref. | 0.98  (0.97, 0.98)  0.98  (0.98, 0.99)  0.97  (0.96, 0.98)  0.94  (0.93, 0.94)  0.86  (0.84, 0.87)  Ref. | 0.97  (0.97, 0.97)  0.98  (0.98, 0.98)  0.96  (0.96, 0.97)  0.94  (0.93, 0.95)  0.87  (0.85, 0.89)  Ref. | 0.98  (0.98, 0.98)  0.99 (  0.98, 0.99)  0.97  (0.96, 0.98)  0.94  (0.93, 0.95)  0.88  (0.86, 0.9)  Ref. | 0.98  (0.98, 0.98)  0.99  (0.98, 0.99)  0.97  (0.96, 0.98)  0.94  (0.93, 0.95)  0.88  (0.86, 0.9)  Ref. |

Note: Matched model 1 used the matched sample, and matched model 2 further adjusted for calendar year and age modeled using restricted cubic splines.

**(b) By birth cohort (follow-up restricted to age 18-22 years)**

|  | **1990-1993 birth cohorts** | | **1994-1996 birth cohorts** | |
| --- | --- | --- | --- | --- |
|  | **Mean income differences (95% CI)** | | | |
|  | **Unmatched** | **Matched** | **Unmatched** | **Matched** |
| Gestational age category  Preterm (24-36 weeks)  Late preterm births (34-36weeks)  Moderately preterm births (32-33 weeks)  Very preterm births (28-31 weeks)  Extremely preterm births (24-27 weeks)  Full-term births (37-41 weeks) | -846 (-921, -771)  -596 (-681, -511)  -942 (-1150, -734)  -1891 (-2134, -1649)  -4409 (-4799, -4019)  Ref. | -605 (-699, -511)  -376 (-485, -268)  -678 (-913, -444)  -1668 (-1939, -1396)  -3896 (-4341, -3451)  Ref. | -1105 (-1185, -1026)  -924 (-1013, -836)  -1033 (-1270, -796)  -1857 (-2137, -1577)  -4682 (-5086, -4279)  Ref. | -613 (-706, -521)  -373 (-473, -273)  -786 (-1057, -515)  -1675 (-2003, -1346)  -4205 (-4674, -3736)  Ref. |
|  | **Ratios of income (95% CI)** | | | |
|  | **Unmatched** | **Matched** | **Unmatched** | **Matched** |
| Gestational age category  Preterm (24-36 weeks)  Late preterm births (34-36weeks)  Moderately preterm births (32-33 weeks)  Very preterm births (28-31 weeks)  Extremely preterm births (24-27 weeks)  Full-term births (37-41 weeks) | 0.93 (0.93, 0.94)  0.95 (0.95, 0.96)  0.93 (0.91, 0.94)  0.85 (0.84, 0.87)  0.66 (0.63, 0.69)  Ref. | 0.95 (0.95, 0.96)  0.97 (0.96, 0.98)  0.95 (0.93, 0.97)  0.87 (0.85, 0.89)  0.69 (0.66, 0.73)  Ref. | 0.92 (0.91, 0.92)  0.93 (0.92, 0.94)  0.92 (0.9, 0.94)  0.86 (0.84, 0.88)  0.64 (0.61, 0.67)  Ref. | 0.95 (0.94, 0.96)  0.97 (0.96, 0.98)  0.94 (0.92, 0.96)  0.87 (0.85, 0.9)  0.67 (0.64, 0.71)  Ref. |
|  | **Risk ratios for employment (95% CI)** | | | |
|  | **Unmatched** | **Matched** | **Unmatched** | **Matched** |
| Gestational age category  Preterm (24-36 weeks)  Late preterm births (34-36weeks)  Moderately preterm births (32-33 weeks)  Very preterm births (28-31 weeks)  Extremely preterm births (24-27 weeks)  Full-term births (37-41 weeks) | 0.95 (0.95, 0.95)  0.96 (0.96, 0.97)  0.94 (0.94, 0.95)  0.89 (0.88, 0.9)  0.78 (0.76, 0.8)  Ref. | 0.97 (0.96, 0.97)  0.98 (0.98, 0.98)  0.96 (0.95, 0.97)  0.91 (0.9, 0.92)  0.81 (0.79, 0.83)  Ref. | 0.94 (0.94, 0.95)  0.95 (0.95, 0.96)  0.94 (0.93, 0.95)  0.89 (0.88, 0.9)  0.77 (0.74, 0.79)  Ref. | 0.96 (0.96, 0.97)  0.97 (0.97, 0.98)  0.95 (0.94, 0.96)  0.91 (0.89, 0.92)  0.8 (0.78, 0.82)  Ref. |
