# Supplementary material for: Association between preterm birth and economic and educational outcomes in adulthood: A population-based matched cohort study
Source: PLoS One. 2024 Nov 6;19(11):e0311895. doi: 10.1371/journal.pone.0311895 (PMC11540172; doi:10.1371/journal.pone.0311895)
Supplement: S6 Table — Associations between preterm birth and (a) postsecondary education enrollment (age 18–22 years) and (b) attainment (age 22–27 years) for individuals born in 1991–1996 in Canada, stratified by birth cohort. (DOCX) [file pone.0311895.s006.docx]

**Association between preterm birth and economic and educational outcomes in adulthood: A population-based matched cohort study**

**Authors:** Asma M. Ahmed, Eleanor Pullenayegum, Sarah D. McDonald, Marc Beltempo, Shahirose S. Premji, Jason D. Pole, Fabiana Bacchini, Prakesh S. Shah, Petros Pechlivanoglou,

**S6 Table. Associations between preterm birth and (a) postsecondary education enrollment (age 18-22 years) and (b) attainment (age 22-27 years) for individuals born in 1991-1996 in Canada, stratified by birth cohort.**

1. **Postsecondary education enrollment (reference category: did not enroll in any postsecondary education)**

|  | **1991-1993 birth cohorts** | | | |
| --- | --- | --- | --- | --- |
|  | **College** | | **University** | |
|  | **Unmatched** | **Matched** | **Unmatched** | **Matched** |
| Gestational age category  Preterm (24-36 weeks)  Late preterm births (34-36weeks)  Moderately preterm births (32-33 weeks)  Very preterm births (28-31 weeks)  Extremely preterm births (24-27 weeks)  Full-term births (37-41 weeks) | 0.9 (0.89, 0.92)  0.92 (0.9, 0.94)  0.88 (0.83, 0.93)  0.87 (0.82, 0.93)  0.81 (0.72, 0.9)  Ref. | 0.94 (0.91, 0.96)  0.94 (0.92, 0.96)  0.91 (0.86, 0.97)  0.94 (0.87, 1.01)  0.88 (0.78, 0.99)  Ref. | 0.81 (0.8, 0.83)  0.84 (0.83, 0.86)  0.75 (0.71, 0.8)  0.7 (0.66, 0.74)  0.54 (0.48, 0.6)  Ref. | 0.84 (0.82, 0.85)  0.87 (0.85, 0.89)  0.79 (0.74, 0.84)  0.73 (0.68, 0.79)  0.56 (0.49, 0.63)  Ref. |
|  | **1994-1996 birth cohorts** | | | |
|  | **College** | | **University** | |
|  | **Unmatched** | **Matched** | **Unmatched** | **Matched** |
| Gestational age category  Preterm (24-36 weeks)  Late preterm births (34-36weeks)  Moderately preterm births (32-33 weeks)  Very preterm births (28-31 weeks)  Extremely preterm births (24-27 weeks)  Full-term births (37-41 weeks) | 0.92 (0.91, 0.94)  0.93 (0.91, 0.95)  0.94 (0.89, 1)  0.85 (0.79, 0.91)  0.82 (0.74, 0.92)  Re. | 0.92 (0.9, 0.94)  0.92 (0.9, 0.95)  0.94 (0.89, 1.00)  0.89 (0.83, 0.96)  0.88 (0.78, 0.99)  Ref. | 0.84 (0.83, 0.86)  0.87 (0.86, 0.89)  0.83 (0.79, 0.88)  0.68 (0.64, 0.72)  0.51 (0.46, 0.57)  Ref. | 0.81 (0.8, 0.83)  0.83 (0.81, 0.85)  0.83 (0.78, 0.88)  0.71 (0.66, 0.77)  0.54 (0.48, 0.62)  Ref. |

1. **Postsecondary education attainment (reference category: did not graduate from any postsecondary education)**

|  | **1991-1993 birth cohorts** | | | | | |
| --- | --- | --- | --- | --- | --- | --- |
|  | **Non-University** | | **University** | | **Postgraduate** | |
|  | **Unmatched** | **Matched** | **Unmatched** | **Matched** | **Unmatched** | **Matched** |
| Gestational age category  Preterm (24-36 weeks)  34-36weeks  32-33 weeks  28-31 weeks  24-27 weeks  Full-term (37-41 weeks) | 0.89 (0.87, 0.91)  0.89 (0.87, 0.91)  0.88 (0.83, 0.94)  0.87 (0.82, 0.94)  0.86 (0.77, 0.97)  Ref. | 0.94 (0.92, 0.96)  0.94 (0.91, 0.96)  0.96 (0.9, 1.03)  0.96 (0.88, 1.03)  0.92 (0.81, 1.05)  Ref. | 0.83 (0.81, 0.84)  0.85 (0.83, 0.87)  0.8 (0.75, 0.84)  0.71 (0.66, 0.76)  0.59 (0.52, 0.67)  Ref. | 0.85 (0.83, 0.87)  0.87 (0.85, 0.89)  0.84 (0.79, 0.89)  0.74 (0.69, 0.8)  0.56 (0.49, 0.65)  Ref. | 0.81 (0.78, 0.85)  0.85 (0.81, 0.89)  0.79 (0.7, 0.9)  0.63 (0.54, 0.74)  0.44 (0.31, 0.61)  Ref. | 0.85 (0.81, 0.89)  0.88 (0.84, 0.93)  0.86 (0.75, 0.99)  0.64 (0.54, 0.76)  0.47 (0.33, 0.68)  Ref. |
|  | **1994-1996 birth cohorts** | | | | | |
|  | **Non-University** | | **University** | | **Postgraduate** | |
|  | **Unmatched** | **Matched** | **Unmatched** | **Matched** | **Unmatched** | **Matched** |
| Gestational age category  Preterm (24-36 weeks)  34-36weeks  32-33 weeks  28-31 weeks  24-27 weeks  Full-term (37-41 weeks) | 0.9 (0.88, 0.91)  0.91 (0.89, 0.93)  0.88 (0.83, 0.93)  0.86 (0.81, 0.92)  0.78 (0.7, 0.88)  Ref. | 0.97 (0.94, 0.99)  0.97 (0.95, 0.99)  0.94 (0.89, 1)  0.93 (0.86, 1.01)  0.96 (0.84, 1.08)  Ref. | 0.86 (0.84, 0.88)  0.89 (0.87, 0.91)  0.85 (0.8, 0.91)  0.72 (0.66, 0.78)  0.5 (0.43, 0.58)  Ref. | 0.84 (0.82, 0.86)  0.86 (0.83, 0.88)  0.85 (0.79, 0.9)  0.74 (0.68, 0.81)  0.53 (0.45, 0.62)  Ref. | 0.79 (0.72, 0.87)  0.82 (0.75, 0.91)  0.81 (0.62, 1.05)  0.66 (0.46, 0.93)  0.27 (0.11, 0.65)  Ref. | 0.85 (0.77, 0.94)  0.87 (0.78, 0.97)  0.89 (0.67, 1.18)  0.76 (0.52, 1.11)  0.37 (0.15, 0.91)  Ref. |
