# Supplementary material for: Association between preterm birth and economic and educational outcomes in adulthood: A population-based matched cohort study
Source: PLoS One. 2024 Nov 6;19(11):e0311895. doi: 10.1371/journal.pone.0311895 (PMC11540172; doi:10.1371/journal.pone.0311895)
Supplement: S7 Table — (DOCX) [file pone.0311895.s007.docx]

**Association between preterm birth and economic and educational outcomes in adulthood: A population-based matched cohort study**

**Authors:** Asma M. Ahmed, Eleanor Pullenayegum, Sarah D. McDonald, Marc Beltempo, Shahirose S. Premji, Jason D. Pole, Fabiana Bacchini, Prakesh S. Shah, Petros Pechlivanoglou,

**S7 Table. Associations between preterm birth and employment income and employment per year, at or after the age of 18 years for individuals born in 1990-1996 in Canada when individuals who died were assigned zero income or unemployed.**

|  | **Mean income differences (95% CI)** | | |
| --- | --- | --- | --- |
|  | **Unmatched** | **Matched model 1** | **Matched model 2** |
| Gestational age category  Preterm (24-36 weeks)  Late preterm births (34-36weeks)  Moderately preterm births (32-33 weeks)  Very preterm births (28-31 weeks)  Extremely preterm births (24-27 weeks)  Full-term births (37-41 weeks) | -2068 (-2138, -1997)  -1481 (-1561, -1401)  -1999 (-2202, -1796)  -3945 (-4174, -3715)  -10101 (-10397, -9806)  Ref. | -1410 (-1516, -1305)  -804 (-930, -678)  -1530 (-1760, -1301)  -3571 (-3833, -3309)  -9296 (-9646, -8946)  Ref. | -1408 (-1512, -1305)  -803 (-926, -679)  -1528 (-1751, -1304)  -3563 (-3820, -3306)  -9280 (-9628, -8932)  Ref. |
|  | **Ratios of income (95% CI)** | | |
|  | **Unmatched** | **Matched model 1** | **Matched model 2** |
| Gestational age category  Preterm (24-36 weeks)  Late preterm births (34-36weeks)  Moderately preterm births (32-33 weeks)  Very preterm births (28-31 weeks)  Extremely preterm births (24-27 weeks)  Full-term births (37-41 weeks) | 0.89 (0.89, 0.89)  0.92 (0.92, 0.92)  0.89 (0.88, 0.9)  0.79 (0.78, 0.8)  0.46 (0.44, 0.47)  Ref. | 0.92 (0.92, 0.93)  0.96 (0.95, 0.96)  0.92 (0.9, 0.93)  0.81 (0.79, 0.82)  0.49 (0.47, 0.51)  Ref. | 0.91 (0.91, 0.92)  0.95 (0.94, 0.96)  0.92 (0.9, 0.93)  0.80 (0.78, 0.82)  0.50 (0.48, 0.52)  Ref. |
|  | **Risk ratios for employment (95% CI)** | | |
|  | **Unmatched** | **Matched model 1** | **Matched model 2** |
| Gestational age category  Preterm (24-36 weeks)  Late preterm births (34-36weeks)  Moderately preterm births (32-33 weeks)  Very preterm births (28-31 weeks)  Extremely preterm births (24-27 weeks)  Full-term births (37-41 weeks) | 0.93 (0.92, 0.93)  0.95 (0.95, 0.96)  0.92 (0.91, 0.93)  0.84 (0.84, 0.85)  0.55 (0.54, 0.56)  Ref. | 0.94 (0.94, 0.95)  0.97 (0.97, 0.97)  0.93 (0.93, 0.94)  0.86 (0.85, 0.87)  0.58 (0.56, 0.59)  Ref. | 0.95 (0.95, 0.96)  0.98 (0.98, 0.98)  0.95 (0.94, 0.95)  0.88 (0.87, 0.89)  0.58 (0.57, 0.60)  Ref. |

Note: Matched model 1 used the matched sample, and matched model 2 further adjusted for calendar year and age modeled using restricted cubic splines.
