# Supplementary material for: Association between preterm birth and economic and educational outcomes in adulthood: A population-based matched cohort study
Source: PLoS One. 2024 Nov 6;19(11):e0311895. doi: 10.1371/journal.pone.0311895 (PMC11540172; doi:10.1371/journal.pone.0311895)
Supplement: S8 Table — Associations between preterm birth and (a) postsecondary education enrollment (age 18–22 years) and (b) attainment (age 22–27 years) for individuals born in 1991–1996 in Canada when individuals who died were categorized as not enrolled/graduated from postsecondary education. (DOCX) [file pone.0311895.s008.docx]

**Association between preterm birth and economic and educational outcomes in adulthood: A population-based matched cohort study**

**Authors:** Asma M. Ahmed, Eleanor Pullenayegum, Sarah D. McDonald, Marc Beltempo, Shahirose S. Premji, Jason D. Pole, Fabiana Bacchini, Prakesh S. Shah, Petros Pechlivanoglou,

**S8 Table. Associations between preterm birth and (a) postsecondary education enrollment (age 18-22 years) and (b) attainment (age 22-27 years) for individuals born in 1991-1996 in Canada when individuals who died were categorized as not enrolled/graduated from postsecondary education.**

1. **Postsecondary education enrollment (reference category: did not enroll in any postsecondary education)**

|  | **Risk ratios for postsecondary education enrollment (95% CI)** | | | |
| --- | --- | --- | --- | --- |
|  | **College** | | **University** | |
|  | **Unmatched** | **Matched** | **Unmatched** | **Matched** |
| Gestational age category  Preterm (24-36 weeks)  Late preterm births (34-36weeks)  Moderately preterm births (32-33 weeks)  Very preterm births (28-31 weeks)  Extremely preterm births (24-27 weeks)  Full-term births (37-41 weeks) | 0.85 (0.84, 0.87)  0.91 (0.89, 0.92)  0.85 (0.81, 0.88)  0.73 (0.7, 0.77)  0.41 (0.38, 0.44)  Ref. | 0.87 (0.85, 0.88)  0.91 (0.9, 0.93)  0.86 (0.82, 0.9)  0.78 (0.75, 0.83)  0.45 (0.42, 0.49)  Ref. | 0.77 (0.76, 0.78)  0.84 (0.83, 0.85)  0.74 (0.71, 0.76)  0.59 (0.56, 0.61)  0.26 (0.24, 0.28)  Ref. | 0.77 (0.76, 0.78)  0.83 (0.81, 0.84)  0.75 (0.72, 0.78)  0.62 (0.59, 0.65)  0.28 (0.26, 0.31)  Ref. |

1. **Postsecondary education attainment (reference category: did not graduate from any postsecondary education)**

|  | **Risk ratios for postsecondary education attainment (95% CI)** | | | | | |
| --- | --- | --- | --- | --- | --- | --- |
|  | **Non-University** | | **University** | | **Postgraduate** | |
|  | **Unmatched** | **Matched** | **Unmatched** | **Matched** | **Unmatched** | **Matched** |
| Gestational age category  Preterm (24-36 weeks)  Late preterm (34-36weeks)  Moderately preterm (32-33 weeks)  Very preterm (28-31 weeks)  Extremely preterm (24-27 weeks)  Full-term (37-41 weeks) | 0.86 (0.84, 0.87)  0.89 (0.87, 0.9)  0.84 (0.81, 0.88)  0.78 (0.75, 0.82)  0.5 (0.46, 0.54)  Ref. | 0.92 (0.9, 0.93)  0.94 (0.93, 0.96)  0.91 (0.87, 0.95)  0.85 (0.81, 0.9)  0.58 (0.53, 0.63)  Ref. | 0.8 (0.79, 0.81)  0.85 (0.83, 0.86)  0.78 (0.75, 0.81)  0.64 (0.61, 0.68)  0.33 (0.3, 0.36)  Ref. | 0.81 (0.8, 0.82)  0.85 (0.84, 0.87)  0.81 (0.77, 0.84)  0.67 (0.64, 0.71)  0.34 (0.31, 0.37)  Ref. | 0.75 (0.72, 0.78)  0.8 (0.77, 0.84)  0.74 (0.66, 0.83)  0.57 (0.49, 0.66)  0.24 (0.18, 0.32)  Ref. | 0.82 (0.79, 0.86)  0.87 (0.83, 0.92)  0.83 (0.73, 0.94)  0.6 (0.51, 0.71)  0.29 (0.21, 0.4)  Ref. |
