# Supplementary material for: Association between preterm birth and economic and educational outcomes in adulthood: A population-based matched cohort study
Source: PLoS One. 2024 Nov 6;19(11):e0311895. doi: 10.1371/journal.pone.0311895 (PMC11540172; doi:10.1371/journal.pone.0311895)
Supplement: S9 Table — (DOCX) [file pone.0311895.s009.docx]

**Association between preterm birth and economic and educational outcomes in adulthood: A population-based matched cohort study**

**Authors:** Asma M. Ahmed, Eleanor Pullenayegum, Sarah D. McDonald, Marc Beltempo, Shahirose S. Premji, Jason D. Pole, Fabiana Bacchini, Prakesh S. Shah, Petros Pechlivanoglou,

**S9 Table. Associations between preterm birth and employment income per year at or after the age of 18 years for individuals born in 1990-1996 in Canada, excluding individuals with zero employment income.**

|  | **Mean income differences (95% CI)** | | |
| --- | --- | --- | --- |
|  | **Unmatched** | **Matched model 1** | **Matched model 2** |
| Gestational age category  Preterm (24-36 weeks)  Late preterm births (34-36weeks)  Moderately preterm births (32-33 weeks)  Very preterm births (28-31 weeks)  Extremely preterm births (24-27 weeks)  Full-term births (37-41 weeks) | - -1000 (-1075, -926)  -870 (-953, -786)  -816 (-1027, -604)  -1666 (-1912, -1421)  -4119 (-4526, -3712)  Ref. | -573 (-684, -462)  -384 (-517, -252)  -508 (-746, -270)  -1536 (-1815, -1257)  -3755 (-4213, -3298)  Ref. | -670 (-780, -560)  -454 (-585, -324)  -638 (-871, -404)  -1792 (-2070, -1514)  -4287 (-4751, -3823)  Ref. |
|  | **Ratios of income (95% CI)** | | |
|  | **Unmatched** | **Matched model 1** | **Matched model 2** |
| Gestational age category  Preterm (24-36 weeks)  Late preterm births (34-36weeks)  Moderately preterm births (32-33 weeks)  Very preterm births (28-31 weeks)  Extremely preterm births (24-27 weeks)  Full-term births (37-41 weeks) | 0.95 (0.95, 0.96)  0.96 (0.96, 0.96)  0.96 (0.95, 0.97)  0.92 (0.91, 0.93)  0.81 (0.79, 0.83)  Ref. | 0.97 (0.97, 0.98)  0.98 (0.98, 0.99)  0.98 (0.97, 0.99)  0.93 (0.92, 0.94)  0.83 (0.81, 0.85)  Ref. | 0.96 (0.95, 0.97)  0.97 (0.96, 0.98)  0.97 (0.96, 0.99)  0.91 (0.89, 0.93)  0.83 (0.81, 0.86)  Ref. |

Note: Matched model 1 used the matched sample, and matched model 2 further adjusted for calendar year and age modeled using restricted cubic splines.
