# Supplementary material for: Association between preterm birth and economic and educational outcomes in adulthood: A population-based matched cohort study
Source: PLoS One. 2024 Nov 6;19(11):e0311895. doi: 10.1371/journal.pone.0311895 (PMC11540172; doi:10.1371/journal.pone.0311895)
Supplement: S10 Table — (DOCX) [file pone.0311895.s010.docx]

**Association between preterm birth and economic and educational outcomes in adulthood: A population-based matched cohort study**

**Authors:** Asma M. Ahmed, Eleanor Pullenayegum, Sarah D. McDonald, Marc Beltempo, Shahirose S. Premji, Jason D. Pole, Fabiana Bacchini, Prakesh S. Shah, Petros Pechlivanoglou,

**S10 Table. Associations between preterm birth and employment income and employment per year, at or after the age of 18 years for individuals born in 1990-1996 in Canada in the subsample linked to maternal tax with and without matching on maternal income and rural residence.**

|  | **Mean income differences (95% CI)** | | | | |
| --- | --- | --- | --- | --- | --- |
|  |  | **Matched on maternal income and rural residence** | | **Not matched on maternal income and rural residence** | |
|  | **Unmatched** | **Matched model 1** | **Matched model 2** | **Matched model 1** | **Matched model 2** |
| Gestational age category  Preterm (24-36 weeks)  Late preterm (34-36weeks)  Moderately preterm (32-33 weeks)  Very preterm (28-31 weeks)  Extremely preterm (24-27 weeks)  Full-term (37-41 weeks) | -1594 (-1677, -1511)  -1315 (-1407, -1222)  -1703 (-1952, -1454)  -2909 (-3196, -2623)  -6425 (-6887, -5963)  Ref. | -732 (-834, -629)  -453 (-565, -340)  -967 (-1285, -649)  -2188 (-2560, -1817)  -5460 (-6060, -4861)  Ref. | -721 (-821, -622)  -450 (-559, -341)  -948 (-1258, -639)  -2131 (-2495, -1768)  -5253 (-5836, -4670)  Ref. | -823 (-920, -726)  -542 (-646, -437)  -1021 (-1320, -722)  -2244 (-2580, -1908)  -5449 (-5990, -4908)  Ref. | -810 (-904, -716)  -537 (-639, -436)  -998 (-1290, -706)  -2181 (-2509, -1852)  -5214 (-5741, -4688)  Ref. |
|  | **Ratios of income (95% CI)** | | | | |
|  |  | **Matched on maternal income and rural residence** | | **Not matched on maternal income and rural residence** | |
|  | **Unmatched** | **Matched model 1** | **Matched model 2** | **Matched model 1** | **Matched model 2** |
| Gestational age category  Preterm (24-36 weeks)  Late preterm (34-36weeks)  Moderately preterm (32-33 weeks)  Very preterm (28-31 weeks)  Extremely preterm (24-27 weeks)  Full-term (37-41 weeks) | 0.91 (0.9, 0.91)  0.92 (0.92, 0.93)  0.9 (0.89, 0.92)  0.83 (0.82, 0.85)  0.63 (0.61, 0.66)  Ref. | 0.96 (0.95, 0.96)  0.97 (0.97, 0.98)  0.94 (0.92, 0.96)  0.87 (0.85, 0.89)  0.67 (0.64, 0.71)  Ref. | 0.96 (0.95, 0.97)  0.97 (0.96, 0.98)  0.95 (0.92, 0.97)  0.87 (0.85, 0.9)  0.73 (0.69, 0.78)  Ref. | 0.95 (0.95, 0.96)  0.97 (0.96, 0.97)  0.94 (0.92, 0.96)  0.87 (0.85, 0.89)  0.67 (0.64, 0.71)  Ref. | 0.95 (0.95, 0.96)  0.97 (0.96, 0.97)  0.94 (0.92, 0.97)  0.87 (0.85, 0.9)  0.73 (0.70, 0.77)  Ref. |
|  | **Risk ratios for employment (95% CI)** | | | | |
|  |  | **Matched on maternal income and rural residence** | | **Not matched on maternal income and rural residence** | |
|  | **Unmatched** | **Matched model 1** | **Matched model 2** | **Matched model 1** | **Matched model 2** |
| Gestational age category  Preterm (24-36 weeks)  Late preterm (34-36weeks)  Moderately preterm (32-33 weeks)  Very preterm (28-31 weeks)  Extremely preterm (24-27 weeks)  Full-term (37-41 weeks) | 0.95 (0.95, 0.95)  0.96 (0.96, 0.96)  0.94 (0.94, 0.95)  0.9 (0.89, 0.91)  0.78 (0.76, 0.8)  Ref. | 0.97 (0.97, 0.97)  0.98 (0.98, 0.98)  0.96 (0.95, 0.97)  0.92 (0.91, 0.93)  0.81 (0.79, 0.83)  Ref. | 0.98 (0.98, 0.98)  0.99 (0.99, 0.99)  0.97 (0.96, 0.98)  0.94 (0.93, 0.95)  0.82 (0.8, 0.84)  Ref. | 0.97 (0.97, 0.97)  0.98 (0.98, 0.98)  0.96 (0.95, 0.97)  0.92 (0.91, 0.93)  0.81 (0.79, 0.83)  Ref. | 0.98 (0.98, 0.98)  0.99 (0.98, 0.99)  0.97 (0.96, 0.98)  0.94 (0.93, 0.95)  0.82 (0.80, 0.84)  Ref. |

Note: Matched model 1 used the matched sample, and matched model 2 further adjusted for calendar year and age modeled using restricted cubic splines.
