# Supplementary material for: Association between preterm birth and economic and educational outcomes in adulthood: A population-based matched cohort study
Source: PLoS One. 2024 Nov 6;19(11):e0311895. doi: 10.1371/journal.pone.0311895 (PMC11540172; doi:10.1371/journal.pone.0311895)
Supplement: S11 Table — Associations between preterm birth and (a) postsecondary education enrollment (age 18–22 years) and (b) attainment (age 22–27 years) for individuals born in 1991–1996 in Canada in the subsample linked to maternal tax with and without matching on maternal income and rural residence. (DOCX) [file pone.0311895.s011.docx]

**Association between preterm birth and economic and educational outcomes in adulthood: A population-based matched cohort study**

**Authors:** Asma M. Ahmed, Eleanor Pullenayegum, Sarah D. McDonald, Marc Beltempo, Shahirose S. Premji, Jason D. Pole, Fabiana Bacchini, Prakesh S. Shah, Petros Pechlivanoglou,

**S11 Table. Associations between preterm birth and (a) postsecondary education enrollment (age 18-22 years) and (b) attainment (age 22-27 years) for individuals born in 1991-1996 in Canada in the subsample linked to maternal tax with and without matching on maternal income and rural residence.**

1. **Postsecondary education enrollment (reference category: did not enroll in any postsecondary education)**

|  | **Matched on maternal income and rural residence** | | | |
| --- | --- | --- | --- | --- |
|  | **College** | | **University** | |
|  | **Unmatched** | **Matched** | **Unmatched** | **Matched** |
| Gestational age category  Preterm (24-36 weeks)  Late preterm births (34-36weeks)  Moderately preterm births (32-33 weeks)  Very preterm births (28-31 weeks)  Extremely preterm births (24-27 weeks)  Full-term births (37-41 weeks) | 0.92 (0.9, 0.93)  0.93 (0.91, 0.94)  0.94 (0.89, 0.98)  0.85 (0.8, 0.9)  0.79 (0.72, 0.87)  Ref. | 0.94 (0.92, 0.96)  0.94 (0.92, 0.96)  0.95 (0.9, 1.01)  0.95 (0.88, 1.02)  0.88 (0.78, 0.99)  Ref. | 0.83 (0.82, 0.84)  0.86 (0.85, 0.88)  0.81 (0.77, 0.85)  0.66 (0.63, 0.7)  0.51 (0.46, 0.56)  Ref. | 0.86 (0.84, 0.88)  0.88 (0.86, 0.9)  0.86 (0.81, 0.91)  0.76 (0.71, 0.81)  0.57 (0.5, 0.64)  Ref. |
|  | **Not matched on maternal income and rural residence** | | | |
|  | **College** | | **University** | |
|  | **Unmatched** | **Matched** | **Unmatched** | **Matched** |
| Gestational age category  Preterm (24-36 weeks)  Late preterm births (34-36weeks)  Moderately preterm births (32-33 weeks)  Very preterm births (28-31 weeks)  Extremely preterm births (24-27 weeks)  Full-term births (37-41 weeks) | - | 0.92 (0.91, 0.94)  0.92 (0.9, 0.94)  0.95 (0.9, 1)  0.92 (0.86, 0.98)  0.85 (0.76, 0.95)  Ref. | - | 0.82 (0.81, 0.84)  0.84 (0.82, 0.86)  0.82 (0.78, 0.87)  0.72 (0.67, 0.76)  0.54 (0.49, 0.61)  Ref. |

1. **Postsecondary education attainment (reference category: did not graduate from any postsecondary education)**

|  | **Matched on maternal income and rural residence** | | | | | |
| --- | --- | --- | --- | --- | --- | --- |
|  | **Non-University** | | **University** | | **Postgraduate** | |
|  | **Unmatched** | **Matched** | **Unmatched** | **Matched** | **Unmatched** | **Matched** |
| Gestational age category  Preterm (24-36 weeks)  Late preterm (34-36weeks)  Moderately preterm (32-33 weeks)  Very preterm (28-31 weeks)  Extremely preterm (24-27 weeks)  Full-term (37-41 weeks)) | 0.89 (0.88, 0.91)  0.9 (0.88, 0.92)  0.89 (0.84, 0.93)  0.86 (0.81, 0.91)  0.79 (0.72, 0.88)  Ref. | 0.97 (0.95, 0.99)  0.97 (0.95, 0.99)  0.95 (0.9, 1.01)  0.99 (0.92, 1.06)  0.91 (0.8, 1.03)  Ref. | 0.89 (0.87, 0.91)  0.87 (0.85, 0.89)  0.88 (0.82, 0.93)  0.68 (0.64, 0.73)  0.51 (0.45, 0.57)  Ref. | 0.89 (0.87, 0.9)  0.9 (0.88, 0.92)  0.87 (0.82, 0.93)  0.81 (0.74, 0.87)  0.6 (0.52, 0.7)  Ref. | 0.92 (0.86, 0.97)  0.79 (0.75, 0.84)  0.69 (0.59, 0.8)  0.45 (0.36, 0.57)  0.25 (0.15, 0.42)  Ref. | 0.92 (0.87, 0.97)  0.95 (0.89, 1.01)  0.9 (0.75, 1.09)  0.68 (0.52, 0.89)  0.38 (0.21, 0.68)  Ref. |
|  | **Not matched on maternal income and rural residence** | | | | | |
|  | **Non-University** | | **University** | | **Postgraduate** | |
|  | **Unmatched** | **Matched** | **Unmatched** | **Matched** | **Unmatched** | **Matched** |
| Gestational age category  Preterm (24-36 weeks)  Late preterm (34-36weeks)  Moderately preterm (32-33 weeks)  Very preterm (28-31 weeks)  Extremely preterm (24-27 weeks)  Full-term (37-41 weeks) | - | 0.95 (0.93, 0.97)  0.95 (0.93, 0.97)  0.95 (0.9, 1.01)  0.95 (0.89, 1.02)  0.89 (0.8, 1)  Ref. | - | 0.85 (0.84, 0.87)  0.87 (0.85, 0.89)  0.85 (0.81, 0.9)  0.76 (0.71, 0.81)  0.54 (0.47, 0.62)  Ref. | - | 0.86 (0.81, 0.91)  0.88 (0.83, 0.94)  0.89 (0.75, 1.06)  0.63 (0.49, 0.8)  0.38 (0.22, 0.64)  Ref. |
