# Supplementary material for: Association between preterm birth and economic and educational outcomes in adulthood: A population-based matched cohort study
Source: PLoS One. 2024 Nov 6;19(11):e0311895. doi: 10.1371/journal.pone.0311895 (PMC11540172; doi:10.1371/journal.pone.0311895)
Supplement: S12 Table — (DOCX) [file pone.0311895.s012.docx]

**Association between preterm birth and economic and educational outcomes in adulthood: A population-based matched cohort study**

**Authors:** Asma M. Ahmed, Eleanor Pullenayegum, Sarah D. McDonald, Marc Beltempo, Shahirose S. Premji, Jason D. Pole, Fabiana Bacchini, Prakesh S. Shah, Petros Pechlivanoglou,

**S12 Table. Associations between preterm birth and employment income and employment per year, at or after the age of 18 years for individuals born in 1983-1996 in Canada.**

|  | **Mean income differences (95% CI)** | | |
| --- | --- | --- | --- |
|  | **Unmatched** | **Matched model 1** | **Matched model 2** |
| Gestational age category  Preterm (24-36 weeks)  Late preterm births (34-36weeks)  Moderately preterm births (32-33 weeks)  Very preterm births (28-31 weeks)  Extremely preterm births (24-27 weeks)  Full-term births (37-41 weeks) | -2092 (-2162, -2021)  -1705 (-1785, -1624)  -2174 (-2370, -1979)  -3780 (-4002, -3557)  -7768 (-8124, -7412)  Ref. | -1263 (-1354, -1173)  -800 (-903, -697)  -1645 (-1869, -1421)  -3288 (-3540, -3037)  -7003 (-7411, -6595)  Ref. | -1214 (-1299, -1129)  -1605 (-1815, -1394)  -948 (-1258, -639)  -3206 (-3443, -2969)  -6592 (-6979, -6205)  Ref. |
|  | **Ratios of income (95% CI)** | | |
|  | **Unmatched** | **Matched model 1** | **Matched model 2** |
| Gestational age category  Preterm (24-36 weeks)  Late preterm births (34-36weeks)  Moderately preterm births (32-33 weeks)  Very preterm births (28-31 weeks)  Extremely preterm births (24-27 weeks)  Full-term births (37-41 weeks) | 0.91 (0.91, 0.91)  0.93 (0.92, 0.93)  0.91 (0.9, 0.92)  0.84 (0.83, 0.85)  0.67 (0.65, 0.68)  Ref. | 0.94 (0.94, 0.95)  0.96 (0.96, 0.97)  0.93 (0.92, 0.94)  0.86 (0.85, 0.87)  0.7 (0.68, 0.71)  Ref. | 0.94 (0.93, 0.94)  0.96 (0.95, 0.97)  0.92 (0.9, 0.93)  0.84 (0.82, 0.85)  0.71 (0.68, 0.73)  Ref. |
|  | **Risk ratios for employment (95% CI)** | | |
|  | **Unmatched** | **Matched model 1** | **Matched model 2** |
| Gestational age category  Preterm (24-36 weeks)  Late preterm births (34-36weeks)  Moderately preterm births (32-33 weeks)  Very preterm births (28-31 weeks)  Extremely preterm births (24-27 weeks)  Full-term births (37-41 weeks) | 0.96 (0.95, 0.96)  0.97 (0.97, 0.97)  0.95 (0.95, 0.95)  0.91 (0.91, 0.92)  0.82 (0.81, 0.83)  Ref. | 0.97 (0.97, 0.97)  0.98 (0.98, 0.98)  0.96 (0.96, 0.97)  0.93 (0.92, 0.93)  0.84 (0.83, 0.85)  Ref. | 0.98 (0.98, 0.98)  0.99 (0.99, 0.99)  0.97 (0.97, 0.98)  0.94 (0.94, 0.95)  0.87 (0.86, 0.88)  Ref. |

Note: Matched model 1 used the matched sample, and matched model 2 further adjusted for calendar year and age modeled using restricted cubic splines.
